# Tarsal Tunnel Syndrome – a narrative review of the literature

**DOI:** 10.1186/1757-1146-8-S2-P9

**Published:** 2015-09-22

**Authors:** Simon C McSweeney, Matthew Cichero

**Affiliations:** 1 Institute of Health Biomedical Innovation (IHBI), Queensland University of Technology, Brisbane, QLD, 4059, Australia; 2 Southwest Podiatric Surgical Services, Great Western Hospitals NHS Foundation Trust, Swindon, SN3 6BB, United Kingdom

**Keywords:** Tarsal tunnel syndrome, tibial neuralgia

## Background

There are many clinical intervention strategies for treating tarsal tunnel syndrome. The role of conservative versus surgical interventions at various stages of the disease process remains unclear, and there is a need for a structured, step-wise approach in treating patients with this syndrome based on derived empirical evidence. This narrative review attempts to analyse the literature to date, for the long-term purpose of developing a clinical treatment algorithm.

## Process

The literature searches that have been incorporated in compiling a comprehensive review of this condition have included: the Cochrane Neuromuscular Group's Specialised Register, Central Register of Controlled trials and Methodology Register (*Cochrane Library 2013)*, the databases of EMBASE, AMED, MEDLINE, CINAHL, Physiotherapy evidence database (PEDRO), Biomed Central, Science Direct and Trip Database (1972 to the present). Authors and experts within the field of lower-limb orthopaedics were contacted to discuss applicable data. Subject-specific criteria searches utilizing the following key terms were performed across all databases:

tarsal tunnel syndrome, tibial neuralgia, compression neuropathy syndromes, tibial nerve impingement, tarsal tunnel neuropathy, entrapment tibial nerve, posterior tibial neuropathy.

## Findings

This literature review has appraised the clinical significance of tarsal tunnel syndrome, whilst assessing varied management interventions (non-surgical and surgical) for the treatment of this condition in both adults and children. According to our review, there is limited high-level robust evidence to guide and refine the clinical management of tarsal tunnel syndrome. Requirements for small-scaled randomized controlled trials in groups with homogenous etiology are needed to analyze the effectiveness of specific treatment modalities.

## Conclusions

It is necessary that further research endeavours be pursued for the clinical understanding, assessment and treatment of Tarsal tunnel syndrome. Accordingly, a structured approach to managing patients who have been correctly diagnosed with this condition should be formulated on the basis of empirical evidence where possible.

